# Ten Commandments for patient-centred treatment

**DOI:** 10.3399/bjgp15X687001

**Published:** 2015-10

**Authors:** Richard Lehman, Aaron M Tejani, James McCormack, Tom Perry, John S Yudkin

**Affiliations:** Senior Advisory Fellow in Primary Care, Cochrane UK, Oxford, UK.; Faculty of Pharmaceutical Sciences, University of British Columbia, Vancouver, Canada.; Professor of Pharmacological Sciences, University of British Columbia, Vancouver, Canada.; Department of Medicine, Health Sciences Mall, University of British Columbia, Vancouver, Canada.; University College London, London, UK.

## 1. Thou shalt have no aim except to help patients, according to the goals they wish to achieve

When deciding on a treatment, the first diagnosis you need to reach is about the nature of the illness. The second diagnosis you need concerns what the individual would like to achieve.^1^ Both are of equal importance and this is as true in simple one-off encounters as in complex lifelong illness. But the balance needs particularly careful thought when beginning long-term treatment.

Always make sure that you understand your patient’s aims before you propose a course of action. It may require 3 minutes in a situation like an acute sore throat, or years of ongoing dialogue in a situation like multiple sclerosis or heart failure. Do not assume that you know what your patient has come for, and do not assume that the treatments you have on offer meet the goals of everyone in the same way.

## 2. Thou shalt always seek knowledge of the benefits, harms, and costs of treatment, and share this knowledge at all times

Both health professionals and lay people tend to overestimate the benefits of treatments and underestimate their harms. The traditional way to express these is as the number-needed-to-treat (NNT) and the number-needed-to-harm (NNH).

It is important to have a ‘ball-park’ idea of these figures in common clinical situations, but also important to bear in mind their limitations. First, patients mostly find NNTs and NNHs hard to understand.^2^ Second, the numbers do not apply to individuals equally but are just average figures across the populations of clinical trials. Third, people vary widely in how they would balance a given benefit against a given harm.^3^

So we need better ways of a) knowing the true NNT and the NNH in the populations we treat; b) sharing this knowledge with people in ways they can understand; and c) applying this knowledge to the goals and preferences of the individual in front of us.

## 3. Thou shalt, if all else fails or if the evidence is lacking, happily consider watchful waiting as an appropriate course of action

The first commandment assumes that there will be two diagnoses in each consultation. But often there will be more, or none.

Many consultations consist of a complex dialogue of exploration, attempted understanding, and partial uncertainty. Unless there is a clear diagnosis, it is usually better to keep the offer open of another consultation rather than issue a prescription.

Other situations where it is often better not to prescribe include acute self-limiting illnesses where symptomatic treatments are available over the counter (OTC). This also applies to some more chronic conditions such as irritable bowel syndrome and chronic back pain, which characteristically fluctuate and for which prescription-only treatments are usually no more effective than cheap OTC alternatives.

The temptation to prescribe rather than offer a timely reassessment should always be resisted.

## 4. Thou shalt honour balanced sources of knowledge, but thou shalt keep thyself from all who may seek to deceive thee

There is no single reliable, unbiased, and continuously updated source of knowledge about effective treatments that can be shared by patients and health professionals. The closest approximation is Wikipedia, which is also the most widely used global resource, although it lacks the support and infrastructure to be comprehensive in its coverage and updating.^4^

Other resources widely used by patients in the UK include NHS Choices and charitable foundation sites such as the British Heart Foundation and Diabetes UK. Health professionals often also access the *BNF*, NICE guidelines, GP notebook, and the Cochrane Library. But none of these are ideally adapted for shared decision making in the consultation.

Clinicians are also targeted for direct and indirect marketing by the pharmaceutical and devices industry to persuade them that new interventions are more effective than old. This is typically not the case, and they are almost always more expensive. Indirect promotion to the public occurs widely via the news media and sometimes through patient organisations if they accept funding from industry. Shared decision making with patients should rest on clear knowledge of harms and benefits, derived from objective analysis and comparison between the best existing alternatives. All industry-sponsored sources of information should be avoided.^5^

## 5. Thou shalt treat according to level of risk and not to level of risk factor

Treating asymptomatic individuals to prevent future adverse events requires a different and longer process of information sharing than treating uncomplicated acute illness. It needs to be based on more rigorous evidence about benefits and harms, especially as these apply to each individual.

Lifelong intervention should be determined by the importance of the outcome to each person, not by the extrapolated reduction of events in the population as a whole. People vary widely in their attitudes to the avoidance of death, myocardial infarction, stroke, or various kinds of cancer, and also in how they weigh the adverse effects of treatment.^3^

The offer of preventive treatment — whether primary or secondary — needs to be made in the context of these individual goals, which can change over time. It should not be addressed to the reduction of a single risk factor but to the totality of risk for a particular outcome. For example, if a person is interested in avoiding coronary heart disease, risk factors need to be explored individually and then aggregated (approximately) using a scoring system. This can then inform a discussion about the various elements of this risk and how it can be reduced using a variety of non-drug and drug interventions. Moreover, such estimates may come up with curious findings; for example, that a statin might provide the best strategy in a high-risk person with a ‘normal’ LDL-cholesterol level.

All drug interventions to reduce risk factors have potential harms and rarely reduce risk in simple relation to reductions in the risk factor (or surrogate marker).^6^

## 6. Thou shalt not bow down to treatment targets designed by committees, for these are but graven images

Traditionally, elevations of single risk factors such as blood pressure or lipid levels have been labelled hypertension or hyperlipidaemia, and individuals (typically without symptoms) have been urged to take drugs to reduce them to a certain level. The very large NNT for such treatment is often not known by clinicians and seldom discussed with recipients, who now acquire a disease label and become patients, followed up at regular intervals for the rest of their lives.

This traditional model has become embedded in many guidelines and in the (UK’s) Quality and Outcomes Framework. Clinicians are paid for the achievement of a surrogate outcome such as systolic blood pressure, total cholesterol, or glycated haemoglobin. This can act as a disincentive to the essential process of dialogue and shared decision making, which always needs to take precedence over the achievement of externally imposed targets.

## 7. Honour thy older patients, for although they often have the highest risk, they may also have the highest risk of harm from treatment

Age increases the risk of cardiovascular disease and most cancers. This amplifies the apparent effect of risk-reducing treatments, but these can only postpone rather than avert death. The quality of remaining life may often be more important than the duration.

For these reasons it is particularly important with older people to take account of individual preferences in the light of NNTs, possible harms, and the absolute prolongation of life offered by each treatment.

Information from randomised controlled trials is often derived from populations without major comorbidity who are younger than most patients with the condition. Therapeutic decision making in older people with multiple conditions and on multiple drugs is fraught with difficulty, and there is often little evidence on which to base combinations of treatment. So it is essential to establish a clear understanding of what individuals are experiencing while on treatment, and what they would like to achieve; and to be honest about possible benefits, harms, and uncertainties.

## 8. Thou shalt stop any treatment that is not of clear benefit and regularly reassess the need for all treatments and tests

Always consider what drugs you can stop before considering those you can start.^7^

At each consultation with a patient who is taking a drug, consider why they are doing so, and consider yourself responsible for its continuation unless there is another clinician with that responsibility. If the consultation is about something else and you do not have time to do this properly, ensure that it is done on another occasion soon. Do not assume that all is well or that someone else will do it. Patients often do not volunteer adverse effects and clinicians often ignore reminders.

## 9. Thou shalt diligently try to find the best treatment for the individual, because different treatments work for different people

The NNT should be just a starting point for clinicians: they give you a rough idea of the statistical likelihood of a particular treatment having some effect within a trial population or pre-specified subgroup. Patients are right to find them hard to apply to themselves.

For most symptomatic conditions, the NNT simply identifies the treatments that may be worth considering first. Cost as well as efficacy may be a consideration. Thereafter, it may require one or more trials of treatment to find the most effective drug. In some situations, such as neuropathic pain, it may be worth trying drugs with a relatively small statistical likelihood of benefit, if this offers the possibility of success when more likely agents have failed.^8^

## 10. Thou shalt seek to use as few drugs as possible

Before printing off a prescription, consider whether a non-drug intervention might be as, or more, effective. Do not use drugs as a shortcut because alternatives might take more time to explain or be harder to access.

As a general rule when prescribing long-term drugs, it is best to use a single agent and use the lowest dose to start with. This usually provides the best balance between benefit and adverse effects.^9^ For example, in heart failure, the lowest doses of an angiotensin-converting enzyme inhibitor have nearly the same mortality benefit as the highest doses, with a much lower risk of hypotension, hypokalaemia, or syncope. If you do decide to uptitrate, discuss the marginal benefit in full with your patient.

It is sometimes useful to use a combination of low-dose agents, for example, to reduce blood pressure. But be aware of potential harms and adverse interactions. Before you increase a dose or add another agent, make sure that you have given your initial treatment an adequate trial and that your patient is really taking it.
